# Circulating Omentin-1 Levels Are Decreased in Dilated Cardiomyopathy Patients with Overt Heart Failure

**DOI:** 10.1155/2016/6762825

**Published:** 2016-05-24

**Authors:** Ying Huang, Yingzhong Lin, Shumin Zhang, Zhijian Wang, Jianwei Zhang, Chao Chang, Ling Liu, Qingwei Ji, Xiaofei Liu

**Affiliations:** ^1^Department of Cardiology, The People's Hospital of Guangxi Zhuang Autonomous Region, Nanning 530021, China; ^2^Department of Cardiology, The People's Hospital of Juxian, Juxian 276500, China; ^3^Department of Cardiology, Beijing Anzhen Hospital, Capital Medical University, Beijing Institute of Heart, Lung, and Blood Vessel Disease, The Key Laboratory of Remodeling-Related Cardiovascular Disease, Ministry of Education, Beijing 100029, China; ^4^Department of Cardiology, China-Japan Friendship Hospital of Ministry of Health, Beijing 100029, China

## Abstract

*Background*. Recent evidence demonstrated that the circulating levels of omentin-1 are related to the presence of ischemic heart disease and heart failure. However, omentin-1 plasma levels in patients with nonischemic dilated cardiomyopathy (DCM), which is the most common etiology of heart failure, have yet to be investigated.* Methods*. Plasma levels of omentin-1 and adiponectin were measured in 100 patients with DCM and 45 healthy controls.* Results*. Plasma omentin-1 levels significantly decreased in DCM patients compared with the control group, whereas adiponectin levels significantly increased in DCM patients compared with the control group. Plasma omentin-1 levels were negatively correlated with adiponectin (*R* = −0.376, *P* = 0.005), C-reactive protein (CRP) (*R* = −0.320, *P* = 0.001), and N-terminal pro-brain natriuretic peptide (NT-proBNP) (*R* = −0.365, *P* = 0.000) levels as well as left ventricular end-diastolic diameter (LVEDD) (*R* = −0.200, *P* = 0.046) but were positively correlated with left ventricular ejection fraction (LVEF) (*R* = 0.496, *P* = 0.000). Plasma adiponectin levels were positively correlated with CRP (*R* = 0.273, *P* = 0.006) and NT-proBNP (*R* = 0.329, *P* = 0.001) levels but were negatively correlated with fasting glucose (*R* = −0.218, *P* = 0.029) and LVEF (*R* = −0.615, *P* = 0.000) levels. Furthermore, omentin-1 (OR 0.983, 95% CI 0.970 to 0.996; *P* = 0.008) levels were independently associated with the presence of DCM before NT-proBNP was added.* Conclusions*. Omentin-1 is a novel biomarker of DCM.

## 1. Introduction

It is accepted that inflammation contributes to the pathophysiology of dilated cardiomyopathy (DCM), which is a common cause of heart failure (HF) and sudden cardiac death [1–3]. A large body of evidence from clinical and experimental studies demonstrated that inflammatory mediators such as tumor necrosis factor-*α* (TNF-*α*) and interleukin-6 (IL-6) impair that cardiac function, resulting in the enlargement of one or both of the ventricles, systolic dysfunction, and HF onset [4–6]. Moreover, high levels of inflammatory mediators are associated with an unfavorable prognosis in HF secondary to DCM [4–6]. Therefore, regulating the inflammatory response is a potential therapeutic method to prevent and treat DCM [6–8].

In addition to energy storage, adipose tissue secretes a wide variety of bioactive substances called adipokines. These adipokines not only partake in metabolic activities that are closely associated with obesity-related diseases such as diabetes but also participate in inflammation and immune regulation that play a critical role in both obesity- and non-obesity-related diseases such as cancer [9, 10]. Most adipokines such as leptin and resistin are proinflammatory mediators, whereas some adipokines such as omentin and adiponectin are anti-inflammatory mediators. Recently, accumulating evidence has demonstrated that adipokines such as adiponectin, visfatin, and leptin are involved in the pathogenesis of HF, although most patients with advanced heart failure have cachectic syndrome [11–15].

Omentin, which was discovered in 2003, is a novel anti-inflammatory adipokine that plays a pivotal role in adipose differentiation, maturation and metabolism, regulation of the immune response, inflammation, and insulin resistance [16, 17]. Omentin has two homologous isoforms (omentin-1 and omentin-2) and omentin-1 is the main isoform that exists in peripheral blood [18]. Accumulating evidence indicates that omentin is a novel biomarker of many cardiovascular diseases such as coronary artery disease, carotid atherosclerosis, and hypertension [17, 19–21]. In addition, some studies have revealed that omentin may participate in ischemic HF [22, 23]. However, no information is available concerning changes in circulating omentin-1 levels in DCM patients. Therefore, we hypothesized that omentin may be involved in DCM, and we measured the levels of plasma omentin-1 and adiponectin, which is another anti-inflammatory adipokine, in DCM patients with overt HF in the present study.

## 2. Materials and Methods

In total, one hundred consecutive patients diagnosed with DCM were enrolled in the present study. Of the patients, twenty-two were classified according to the standards of the New York Heart Association (NYHA) Classification as functional class II (DCM1 group), 45 patients as class III (DCM2 group), and 33 patients as class IV (DCM3 group); no patients were enrolled as class I. Inclusion criteria were (1) left ventricular ejection fraction (LVEF) less than 40% and left ventricular end-diastolic diameter (LVEDD) more than 55 mm and (2) angiographically normal coronary arteries. The control group consisted of 45 age-matched healthy subjects. The clinical profiles of patients and healthy controls are given in Table 1.

Subjects with hypertension, diabetes, coronary artery disease, congenital or valvular heart disease, myocarditis, and pericarditis were excluded. Specific cardiomyopathy phenotypes such as hypertrophic cardiomyopathy, peripartum cardiomyopathy, and alcoholic cardiomyopathy were also excluded. In addition, patients with advanced liver disease, renal failure, malignant disease, septicemia, current steroid therapy, and other inflammatory diseases were excluded from the study.

Written informed consent was obtained from each patient. The study was approved by the Ethics Committee of China-Japan Friendship Hospital, the People's Hospital of Guangxi Zhuang Autonomous Region, and Beijing Anzhen Hospital.

Fasting blood samples were obtained the morning after admission. Samples were collected in sodium heparin Vacutainers (Becton-Dickinson). Blood was centrifuged for 15 min at 3,000 ×g and the plasma was stored at −80°C until further use.

Plasma omentin-1 and adiponectin (R&D Systems, USA) levels were measured by ELISA following the manufacturer's instructions. ELISA intra-assay and interassay coefficients of variation were <5% and <10%, respectively. All of the samples were measured in duplicate.

Patients underwent M-mode and 2D-echocardiography using a GE ViVid E7 ultrasonography machine (GE Healthcare, USA) with a transthoracic 1.5–4.3 MHz probe (M5S-D). LVEDD and fractional shortening were measured. LVEF was calculated from the apical four-chamber position by the area-length method.

All of the data are presented as the mean ± SD. When comparing only 2 groups, Student's *t*-test was used. For comparisons involving 3 or more groups, a one-way ANOVA followed by the Newman-Keuls post hoc test was used. Spearman's correlation was used to calculate the correlations between plasma adipokine levels and the other measured parameters. Simple linear regression analyses and subsequent binary logistic regression analyses were performed to identify the independent predictors of DCM. The candidate variables entered in the model included age, sex, body mass index (BMI), heart rate (HR), systolic blood pressure (SBP), diastolic blood pressure (DBP), lipid and lipoprotein fractions, fasting glucose, creatinine, creatine kinase MB (CKMB), TnI, C-reactive protein (CRP), N-terminal pro-brain natriuretic peptide (NT-proBNP), and omentin-1 and adiponectin levels. Odds ratios (ORs) and 95% confidence intervals (CIs) were calculated. In all of the tests, a value of *P* < 0.05 was considered to be statistically significant.

## 3. Results

There were no differences in sex, age, and smoking between the control and DCM groups. Patients with DCM displayed lower BMI, total triglycerides (TG) levels and LVEF, and higher HR, DBP, creatinine, CRP, CKMB, TnI, and NT-proBNP levels in addition to LVEDD compared to the control group (Table 1).

**Figure 1:**
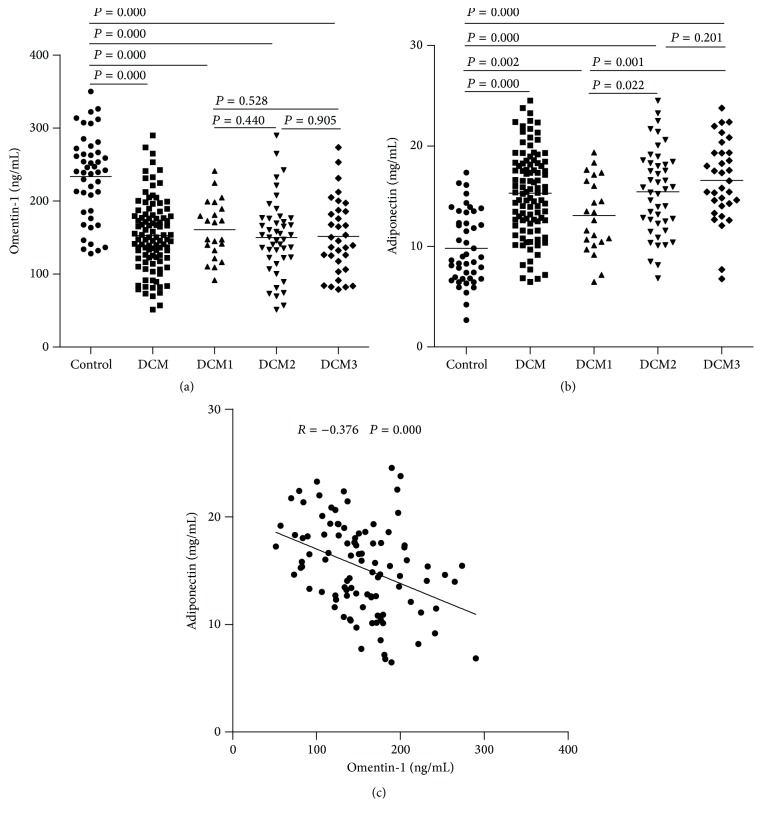
Plasma adipokines concentrations in each group. (a) Plasma omentin-1 levels in patients with DCM were significantly increased compared with the control group. (b) Plasma adiponectin levels in patients with DCM were significantly increased compared with the control group. (c) Plasma omentin-1 levels were negatively correlated with plasma adiponectin levels.

As demonstrated in Table 2 and Figure 1, plasma omentin-1 levels in DCM patients were significantly decreased compared with the control group. Subgroup analysis revealed that plasma omentin-1 levels in the DCM1, DCM2, and DCM3 groups were significantly decreased compared with the control group; however, plasma omentin-1 levels were not different in the DCM1, DCM2, and DCM3 groups. Plasma adiponectin levels in DCM patients were significantly increased compared with the control group. Subgroup analysis demonstrated that plasma adiponectin levels in the DCM1, DCM2, and DCM3 groups were significantly increased compared with the control group; in addition, plasma adiponectin levels in the DCM2 and DCM3 groups were significantly increased compared with the DCM1 group, whereas there were no differences between the DCM2 and DCM3 groups (Figure 1). A correlation analysis demonstrated that plasma omentin-1 levels were negatively correlated with plasma adiponectin levels in DCM patients (Figure 1).

Next, we analyzed whether changes in adipokines levels corresponded with sex, smoking, or medication. As shown in Table 3, circulating omentin-1 and adiponectin levels exhibited no significant differences in their association.

As shown in Table 4, plasma omentin-1 levels were negatively correlated with CRP (*R* = −0.320, *P* = 0.001), NT-proBNP (*R* = −0.365, *P* = 0.000), and LVEDD (*R* = −0.200, *P* = 0.046) levels but were positively correlated with LVEF (*R* = 0.496, *P* = 0.000). Plasma adiponectin levels were positively correlated with CRP (*R* = 0.273, *P* = 0.006) and NT-proBNP (*R* = 0.329, *P* = 0.001) levels but were negatively correlated with fasting glucose (*R* = −0.218, *P* = 0.029) levels and LVEF (*R* = −0.615, *P* = 0.000).

Simple logistic regression analysis demonstrated that BMI, TG, HR, DBP, creatinine, CKMB, TnI, CRP, NT-proBNP, omentin-1, and adiponectin levels exhibited a trend (*P* < 0.05) toward an association with DCM. Binary logistic regression analyses were subsequently performed using a model including the following variables: BMI, TG, HR, DBP, creatinine, CKMB, TnI, CRP, NT-proBNP, omentin-1, and adiponectin. These results demonstrated that omentin-1 (OR 0.983, 95% CI 0.970 to 0.996; *P* = 0.008), HR (OR 1.076, 95% CI 1.016 to 1.140; *P* = 0.013), CRP (OR 7.166, 95% CI 1.551 to 33.098; *P* = 0.012), and TnI (OR 64.078, 95% CI 2.908 to 241.933; *P* = 0.008) were independently associated with DCM before NT-proBNP levels, the gold standard for the diagnosis of heart failure, were added. However, when the NT-proBNP level was added to the analyses, these associations disappeared.

## 4. Discussion

In the present study, we measured the plasma levels of two anti-inflammatory adipokines, omentin-1 and adiponectin, in DCM patients. Consistent with previous studies [11–13], plasma adiponectin levels were significantly increased in DCM patients, and this rise was accompanied with the NYHA class. In contrast, plasma omentin-1 levels were significantly decreased in patients with DCM compared with healthy controls and were negatively correlated with the increased adiponectin levels in DCM. Binary logistic regression analyses also demonstrated that decreased omentin-1 levels were an independent predictor of DCM. Therefore, these results suggested that omentin-1, a novel anti-inflammatory adipokine, plays a role in DCM.

Omentin was initially identified from visceral omental adipose tissue [16]. Insulin, glucose, adiponectin, and proinflammatory mediators such as IL-6 and TNF-*α* promote omentin expression [16–18]. Omentin production is high in the stromal vascular fraction of visceral adipose tissue and is easily detected in human epicardial fat, suggesting a potential role for omentin in cardiovascular disease [24]. Omentin has also been identified in the thymus, small intestine, colon, and reticulocytes, but it is scarcely detectable in subcutaneous fat depots and mature adipocytes. Accumulating evidence has demonstrated that omentin not only acts as an adipocytokine that participates in energy metabolism but also serves as an anti-inflammatory cytokine involved in inflammatory regulation, and it is associated with chronic inflammatory and autoimmune diseases such as diabetes, atherosclerotic disease, and rheumatoid arthritis [16, 17, 19, 20, 25].

Data have shown that decreased omentin levels may be associated with HF secondary to ischemic heart disease [22]. According to the NYHA classification, 59 patients with coronary heart disease were divided into 3 groups: 11 cases with class I as group one, 36 cases with class II/III as group two, and 12 cases with class IV as group three. Wang et al. determined that plasma omentin-1 levels were significantly decreased in groups two and three compared with group one and that plasma omentin-1 levels were significantly decreased in group three compared with group two, suggesting that plasma omentin-1 levels were closely associated with HF severity [22]. However, some limitations exist in that study: the sample was too small and the echocardiography results in those enrolled patients were not provided. In addition, a reason for classification of patients with class II/III into the same group was not given. Taken together, the results from Wang et al. and our studies indicated that circulating omentin-1 levels are significantly decreased in HF regardless of the etiology.

Some studies revealed that medication use regulated adiponectin production in HF patients, whereas some studies did not [26–28]. Thus, we analyzed the effect of medication on omentin-1 and adiponectin levels. These results, however, revealed no difference in the circulating levels of these adipokines based on ACEI or ARB, *β*-blocker, diuretic, digitalis, or spironolactone use. Giannessi et al. determined that adiponectin levels were negatively correlated with BMI and positively correlated with HDL in patients with DCM without overt heart failure, whereas there were no correlations between adiponectin and blood pressure, TC, TG, LDL-C, and fasting glucose [29]. In the present study, adiponectin levels were negatively correlated with fasting glucose levels but were not correlated with age, heart rate, blood pressure, BMI, lipid and lipoprotein fractions, or creatinine in DCM patients. In addition, no correlations between omentin-1 and those parameters were observed in the present study.

Previous studies reported that higher adiponectin levels are associated with an unfavorable prognosis in HF patients [11, 28]. Recently, a small sample study that enrolled 136 consecutive patients with HF and about one year of follow-up indicated that low omentin-1 levels are an independent predictor of cardiac events in those patients [23]. However, the inclusion criteria were significantly defective: baseline clinical characteristics demonstrated that there were no differences in LVEDD between HF patients and control cases, and the mean LVEF in HF patients is 50 ± 18; therefore, according to HF guidelines, some enrolled patients may not have been diagnosed with HF. In the present study, four biomarkers (including CKMB, TnI, CRP, and NT-proBNP) and two left ventricular function parameters (LVEF and LVEDD) were used to indicate DCM severity. We observed that increased adiponectin levels were positively correlated with CRP and NT-proBNP levels and negatively correlated with LVEF. In contrast, decreased omentin-1 levels were positively correlated with LVEF and negatively correlated with CRP and NT-proBNP levels as well as LVEDD. Another clinical study also demonstrated that plasma omentin levels were positively correlated with improved LVEF in AMI patients who had undergone successful reperfusion treatment [30]. Taken together, the results suggested a tight relationship between omentin and heart function. Using a myocardial ischemia/reperfusion (I/R) injury model, Kataoka et al. also demonstrated a protective mechanism of omentin that ameliorated myocardial ischemic damage and apoptosis via blocking AMP-activated protein kinase (AMPK) phosphorylation and Akt activity [30]. However, the exact role of omentin in the development of DCM should be investigated in the future.

In summary, the results of our study are the first to demonstrate that plasma omentin-1 levels were dramatically decreased in DCM and that decreased plasma omentin-1 levels were associated with inflammation and left ventricular function in DCM. More importantly, omentin-1 levels were independently associated with DCM before NT-proBNP was added. Thus, omentin-1 may serve as a novel biomarker of DCM. However, the present study has some limitations. In most situations, DCM patients with class I do not go to hospital because of no symptoms, although their heart function has been impaired. Therefore, we fail to enroll these patients in the present study. Whether plasma omentin-1 levels are elevated in DCM patients with class I remains uncertain, which should be investigated in the future.

## Figures and Tables

**Table 1:** Clinical characteristics of patients.

Characteristics	Control	DCM	DCM1	DCM2	DCM3
Age (years)	52 ± 12	54 ± 13	58 ± 11	54 ± 13	52 ± 15
Sex (male/female)	25/20	72/28	14/8	32/13	26/7
Smoking, *n* (%)	10 (22.2)	24 (24.0)	2 (9.1)	13 (28.9)	9 (27.3)
BMI (Kg/m^2^)	25.6 ± 3.4	23.9 ± 3.8^*∗*^	23.4 ± 3.2^*∗*^	23.9 ± 3.2	24.3 ± 4.9
HR (bpm)	71 ± 10	90 ± 18^*∗*^	89 ± 19^*∗*^	87 ± 17^*∗*^	93 ± 19^*∗*^
SBP (mmHg)	122 ± 12	119 ± 15	124 ± 13	119 ± 14	116 ± 17
DBP (mmHg)	71 ± 9	76 ± 12^*∗*^	76 ± 9	75 ± 12	76 ± 15
TG (mmol/L)	1.57 ± 0.85	1.28 ± 0.73^*∗*^	1.09 ± 0.33	1.46 ± 0.95	1.16 ± 0.54
TC (mmol/L)	4.51 ± 0.98	4.19 ± 1.09	4.14 ± 1.31	4.31 ± 1.14	4.09 ± 0.86
HDL-C (mmol/L)	1.15 ± 0.29	1.06 ± 0.34	1.19 ± 0.40	1.08 ± 0.31	0.94 ± 0.30^*∗*^
LDL-C (mmol/L)	2.72 ± 0.80	2.70 ± 0.85	2.40 ± 0.70	2.83 ± 1.02	2.73 ± 0.62
GLU (mmol/L)	5.08 ± 0.54	4.95 ± 1.23	5.15 ± 1.55	4.80 ± 0.83	5.01 ± 1.44
Creatinine (*µ*mol/L)	72.46 ± 18.80	100.23 ± 41.43^*∗*^	104.68 ± 69.54^*∗*^	98.84 ± 31.74^*∗*^	99.15 ± 27.09^*∗*^
CRP (mg/L)	0.87 ± 0.65	7.71 ± 8.69^*∗*^	6.07 ± 6.79^*∗*^	6.93 ± 7.99^*∗*^	9.87 ± 10.38^*∗*^
CKMB (ng/mL)	1.27 ± 0.81	2.15 ± 1.50^*∗*^	1.84 ± 1.18	2.15 ± 1.34^*∗*^	2.36 ± 1.85^*∗*^
TnI (ng/mL)	0.01 ± 0.01	0.06 ± 0.18^*∗*^	0.04 ± 0.06^*∗*^	0.09 ± 0.26^*∗*^	0.04 ± 0.04^*∗*^
NT-proBNP (pg/mL)	94 ± 84	5053 ± 5590^*∗*^	3513 ± 3074^*∗*^	4980 ± 6092^*∗*^	6179 ± 6040^*∗*^
LVEF (%)	65.33 ± 5.67	29.89 ± 5.94^*∗*^	32.41 ± 4.89^*∗*^	30.20 ± 5.65^*∗*^	27.79 ± 6.36^*∗*^
LVEDD (mm)	46.91 ± 3.60	65.95 ± 7.52^*∗*^	65.27 ± 7.35^*∗*^	66.09 ± 7.39^*∗*^	66.21 ± 7.99^*∗*^
Medications, *n* (%)					
ACEI/ARB	0	37 (37)	10 (45.4)	11 (24.4)	16 (48.5)
*β*-blocker	0	19 (19)	8 (36.4)	7 (15.6)	4 (12.1)
Diuretics	0	46 (46)	11 (50)	14 (31.1)	21 (63.6)
Digitalis	0	44 (44.0)	10 (45.4)	13 (28.9)	21 (63.6)
Spironolactone	0	31 (31)	7 (31.8)	12 (26.7)	12 (36.4)

The data are given as the mean ± SD or number of patients. DCM: dilated cardiomyopathy; BMI: body mass index; HR: heart rate; SBP: systolic blood pressure; DBP: diastolic blood pressure; TG: total triglycerides; TC: total cholesterol; HDL-C: high-density lipoprotein cholesterol; LDL-C: low-density lipoprotein cholesterol; GLU: fasting glucose; CRP: C-reactive protein; NT-proBNP: N-terminal pro-brain natriuretic peptide; LVEF: left ventricular ejection fraction; LVEDD: left ventricular end-diastolic dimension; ACEI: angiotensin-converting enzyme inhibitor; ARB: angiotensin receptor blocker.

^*∗*^ *P* < 0.05 versus control.

**Table 2:** Plasma levels of adipokines in each group.

	Number	Omentin-1 (ng/mL)	Adiponectin (mg/mL)
Control	45	233.33 ± 58.04	9.81 ± 3.55
DCM	100	153.00 ± 48.94^*∗∗*^	15.30 ± 4.20^*∗∗*^
DCM1	22	163.99 ± 53.73^*∗∗*^	13.09 ± 3.68^*∗∗*^
DCM2	45	150.21 ± 52.02^*∗∗*^	15.44 ± 4.23^*∗∗*,#^
DCM3	33	151.65 ± 51.01^*∗∗*^	16.59 ± 4.01^*∗∗*,##^

Note: the data are given as the mean ± SD. ^*∗∗*^ *P* < 0.01 versus control, ^#^ *P* < 0.05 versus DCM1, and ^##^ *P* < 0.01 versus DCM1.

**Table 3:** Plasma levels of adipokines according to disease status and prescription medication history in DCM.

	Number	Omentin-1 (ng/mL)	Adiponectin (mg/mL)
Sex			
Male	72	149.67 ± 46.20	15.31 ± 4.10
Female	28	161.56 ± 55.36	15.29 ± 4.54
Smoking			
Yes	24	143.04 ± 50.10	15.27 ± 4.27
No	76	156.14 ± 48.48	15.42 ± 4.05
ACEI/ARB			
Yes	37	150.45 ± 52.30	15.98 ± 3.98
No	63	154.50 ± 47.22	15.43 ± 4.36
*β*-blocker			
Yes	19	145.49 ± 40.09	15.56 ± 3.95
No	81	154.76 ± 50.85	15.24 ± 4.28
Diuretics			
Yes	46	152.18 ± 50.60	14.81 ± 3.97
No	54	153.69 ± 47.94	15.72 ± 4.38
Digitalis			
Yes	44	155.04 ± 49.39	14.99 ± 3.97
No	56	151.39 ± 48.97	15.55 ± 4.40
Spironolactone			
Yes	31	152.48 ± 56.70	14.59 ± 3.98
No	69	153.23 ± 45.48	15.67 ± 4.29

Note: the data are given as the mean ± SD.

**Table 4:** Correlation analysis in DCM.

Characteristics	Omentin-1 (ng/mL)	Adiponectin (mg/mL)
Age (years)	0.068	−0.047
BMI (Kg/m^2^)	−0.038	−0.144
HR (bpm)	−0.134	−0.048
SBP (mmHg)	0.016	−0.143
DBP (mmHg)	−0.019	−0.007
TG (mmol/L)	−0.006	0.005
TC (mmol/L)	−0.071	0.013
HDL-C (mmol/L)	0.041	−0.094
LDL-C (mmol/L)	−0.090	0.034
GLU (mmol/L)	0.089	−0.218^*∗*^
Creatinine (*µ*mol/L)	0.130	0.188
CKMB (ng/mL)	−0.002	−0.045
TnI (ng/mL)	−0.002	0.088
CRP (mg/L)	−0.320^*∗∗*^	0.273^*∗∗*^
NT-proBNP (pg/mL)	−0.365^*∗∗*^	0.329^*∗∗*^
LVEF (%)	0.496^*∗∗*^	−0.615^*∗∗*^
LVEDD (mm)	−0.200^*∗*^	−0.003

Note: ^*∗*^ *P* < 0.05 and ^*∗∗*^ *P* < 0.01.
